# Abnormal brain development of monoamine oxidase mutant zebrafish and impaired social interaction of heterozygous fish

**DOI:** 10.1242/dmm.049133

**Published:** 2022-02-23

**Authors:** Diego Baronio, Yu-Chia Chen, Pertti Panula

**Affiliations:** Department of Anatomy, University of Helsinki, 00014 U Helsinki, Finland

**Keywords:** Dopamine, Serotonin, Histamine, Monoamine oxidase, Autism

## Abstract

Monoamine oxidase (MAO) deficiency and imbalanced levels of brain monoamines have been associated with developmental delay, neuropsychiatric disorders and aggressive behavior. Animal models are valuable tools to gain mechanistic insight into outcomes associated with MAO deficiency. Here, we report a novel genetic model to study the effects of *mao* loss of function in zebrafish. Quantitative PCR, *in situ* hybridization and immunocytochemistry were used to study neurotransmitter systems and expression of relevant genes for brain development in zebrafish *mao* mutants. Larval and adult fish behavior was evaluated through different tests. Stronger serotonin immunoreactivity was detected in *mao*^+/−^ and *mao*^−/−^ larvae compared with their *mao*^+/+^ siblings. *mao*^−/−^ larvae were hypoactive, and presented decreased reactions to visual and acoustic stimuli. They also had impaired histaminergic and dopaminergic systems, abnormal expression of developmental markers and died within 20 days post-fertilization. *mao*^+/−^ fish were viable, grew until adulthood, and demonstrated anxiety-like behavior and impaired social interactions compared with adult *mao*^+/+^ siblings. Our results indicate that *mao*^−/−^ and *mao*^+/−^ mutants could be promising tools to study the roles of MAO in brain development and behavior.

This article has an associated First Person interview with the first author of the paper.

## Abstract (precis)

**Summary:** We assessed developmental, neurochemical and behavioral alterations displayed by *mao^+/−^* and *mao*^−/−^ zebrafish, establishing that these model organisms are promising tools to study the consequences of *MAOA/B* deficiency.

## INTRODUCTION

Monoamine oxidases catalyze the oxidative deamination of several endogenous and dietary amines, including dopamine (DA), norepinephrine (NE), serotonin (5-HT) and the first histamine metabolite, tele-N-methylhistamine. In mammals, there are two isoforms (MAOA and MAOB), which can be distinguished on the basis of their substrate specificity and their sensitivity towards specific inhibitors. MAOA deaminates 5-HT and NE, whereas MAOB deaminates phenethylamine (PEA) (Glover et al., 1977). DA is primarily metabolized by MAOA in rodents and by MAOB in humans (Bortolato et al., 2008). Clorgyline and deprenyl inhibit MAOA and MAOB, respectively (Sullivan et al., 1986).

Several studies link both MAO forms to different brain disorders (Jones and Raghanti, 2021). A role for increased MAOA activity/expression in the pathophysiology of different forms of depression, such as major depressive disorder and postpartum depression, has been established (Sacher et al., 2010; Schulze et al., 2000). A complete and selective deficiency of enzymatic activity of MAOA was described in Brunner syndrome patients (Brunner et al., 1993). They presented antisocial and violent conduct as a maladaptive response to environmental triggers, mild cognitive impairments, stereotyped hand movements and parasomnias. Some reports indicate a role for MAOA in the pathophysiology of autism spectrum disorder (ASD) (Bortolato et al., 2018). Particularly, low-activity *MAOA* variants have been linked to higher severity of social impairment in boys with ASD (Cohen et al., 2011). Postmortem brain tissue from subjects with ASD were analyzed, and a significant impairment in the activity of MAOA in the cerebellum and frontal cortex was detected (Gu et al., 2017). *Maoa* loss of function in mice leads to high levels of brain 5-HT and NE and aggressiveness (Cases et al., 1995). *Maoa* knockout (KO) mice also recapitulate the main core deficits observed in ASD, including social impairment and communication impairments (Bortolato et al., 2013). Additionally, *Maoa* mutant mice display stereotypic behavior across several tasks, including marble burying, hole-board exploration and spontaneous alternations in a T maze (Bortolato et al., 2013).

The single-nucleotide polymorphism (SNP) rs1799836 of *MAOB* was selected for association analysis in 537 schizophrenia patients and 536 healthy controls, and it was identified as a risk factor in the development of schizophrenia (Wei et al., 2011). In Alzheimer's disease, MAOB has been found to be increased in astrocytes and pyramidal neurons. Additionally, it plays a key role in amyloid β-peptide formation (Schedin-Weiss et al., 2017). A strong association between the *MAOB* gene and adult attention deficit hyperactivity disorder (ADHD) was also identified (Ribasés et al., 2009), and reports indicate that deprenyl reduces ADHD symptoms. This effect of MAOB inhibition on ADHD symptoms may lie in the increased level of PEA, which is assumed to act as an endogenous amphetamine (Janssen et al., 1999). Interestingly, *Maob* mutant mice display increased brain levels of PEA, but unaltered locomotor activity, and do not display anxious-like behavior and, differently from *Maoa* mutant mice, do not show aggressive behavior (Grimsby et al., 1997).

Zebrafish has become a popular model organism in neuroscience because its genes share homology with those of humans and it possesses all main neurotransmitters (Gerlai, 2011; Kalueff et al., 2014; Panula et al., 2010). Unlike mammals, zebrafish possess only one form of *mao*, which displays a strong affinity for 5-HT and a modest one for DA, and is inhibited by both clorgyline and deprenyl (Anichtchik et al., 2006). To our knowledge, a *mao* zebrafish mutant has not been described and characterized yet. The study of outcomes related to disruption of Mao functioning in zebrafish development has been limited to the use of pharmacological inhibitors (He et al., 2013; Sallinen et al., 2009b; Setini et al., 2005). Although this approach is widely used and can provide valuable information, interpreting the results of zebrafish exposure to chemicals, more specifically at early development, can be challenging for several reasons. For example, there are wide variations in the duration of exposure, whether the animals are exposed individually or in groups, whether the chemical is renewed daily or not, the ‘window’ of exposure, and how soon after exposure the animal is assessed. Moreover, deprenyl can be metabolized to amphetamine, which could alter DA neurotransmission and generate effects independent of Mao inhibition (Karoum et al., 1982).

Here, we analyzed the monoaminergic systems, developmental markers and behavioral parameters of larval *mao*^−/−^ and *mao*^+/−^ zebrafish in comparison with those of their *mao*^+/+^ siblings. Furthermore, in adult and juvenile *mao*^+/−^ zebrafish, we analyzed parameters relevant for ASD, such as social behavior, anxiety, Mao activity and gene expression.

## RESULTS

### Larval behavior and serotonergic system

**Fig. 1.**
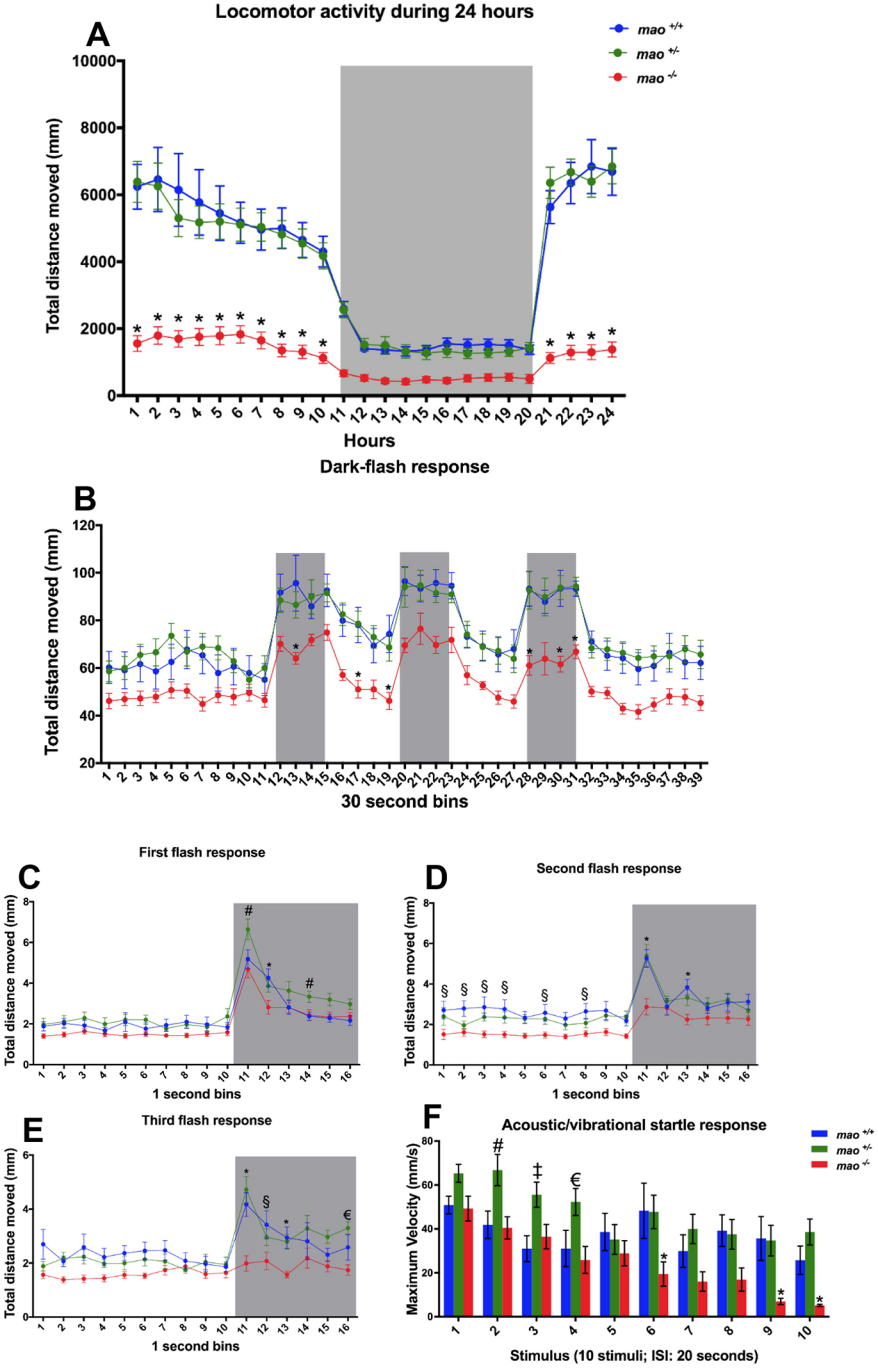
**Behavioral phenotype of *mao* mutants.** (A) *mao*^−/−^ larvae were hypoactive during diurnal and nocturnal (gray shaded area) locomotor activity tracking at 6 dpf. *n*=16 for *mao*^+/+^, *n*=16 for *mao*^+/−^ and *n*=15 for *mao*^−/−^. (B) Larvae at 6 dpf were exposed to alternating 2 min periods of light and darkness (gray shaded areas), with three periods of darkness in total. *n*=15 *mao*^+/+^ and *n*=16 for *mao*^+/−^ and *mao*^−/−^. (C-E) Analysis of the larval response to sudden darkness [first (C), second (D) and third (E) flash response] in 1 s bins. Gray shaded areas indicate periods of darkness. (F) Mean values for maximum velocity during ten acoustic/vibrational stimuli with a 20 s interstimulus interval (ISI). *n*=15 for *mao*^+/+^, *n*=16 for *mao*^+/−^ and *n*=12 for *mao*^−/−^. Two-way ANOVA followed by Tukey's post hoc test was used for statistical analysis. ^#^*P*<0.05 (*mao*^+/−^ versus *mao*^+/+^ and *mao*^−/−^), *P<0.05 (*mao*^+/+^ and *mao*^+/−^ versus *mao*^−/−^), ^§^*P*<0.05 (*mao*^+/+^ versus *mao*^−/−^), ^€^*P*<0.05 (*mao*^+/−^ versus *mao*^−/−^), ^‡^*P*<0.05 (*mao*^+/−^ versus *mao*^+/+^).

*mao*^−/−^ larvae [6 days post-fertilization (dpf)] were hypoactive during a 24 h tracking period [*n*=16 for *mao*^+/+^, *n*=16 for *mao*^+/−^ and *n*=15 for *mao*^−/−^; two-way ANOVA, genotype effect *F*(2, 1056)=352.6, *P*<0.01; Tukey's post hoc test significances indicated in the graph; Fig. 1A]. *mao*^+/−^ larvae displayed a similar pattern of activity to their *mao*^+/+^ siblings. During the dark-flash behavioral test (6 dpf), *mao*^−/−^ larvae also showed signs of hypoactivity in comparison with *mao*^+/+^ and *mao*^+/−^ siblings [*n*=15 *mao*^+/+^ and *n*=16 for *mao*^+/−^ and *mao*^−/−^; two-way ANOVA, genotype effect *F*(2, 1716)=174.3, *P*<0.01; Tukey's multiple comparisons test significances indicated in the graph; Fig. 1B], whereas *mao*^+/+^ and *mao*^+/−^ larvae displayed similar behavior in an analysis of 30 s bins. Interestingly, when we analyzed the larval response to sudden darkness in 1 s bins, *mao*^+/−^ larvae displayed a significantly stronger reaction than *mao*^+/+^ and *mao*^−/−^ larvae in the first dark flash. Additionally, compared with *mao*^+/+^ larvae, *mao*^+/−^ larvae took longer to adapt and return to the baseline activity [two-way ANOVA, genotype effect *F*(2, 704)=30.4, *P*<0.01; Tukey's post hoc test significances indicated in the graph; Fig. 1C]. During the subsequent stimuli, no differences between *mao*^+/+^ and *mao*^+/−^ larvae were detected, with both genotypes responding significantly stronger than *mao*^−/−^ larvae at the onset of darkness in the second [two-way ANOVA, genotype effect *F*(2, 704)=49.89, *P*<0.01; Tukey's post hoc test significances indicated in the graph; Fig. 1D] and third [two-way ANOVA, genotype effect *F*(2, 704)=41.44, *P*<0.01; Tukey's post hoc test significances indicated in the graph; Fig. 1E] flashes.

In the acoustic/vibrational startle test (at 6 dpf), we observed that the response after the first stimulus did not differ between *mao*^+/+^, *mao*^+/−^ and *mao*^−/−^ larvae [*n*=15 *mao*^+/+^, *n*=16 for *mao*^+/−^ and *n*=12 for *mao*^−/−^; two-way ANOVA, genotype effect *F*(2, 400)=26.96, *P*<0.01; Tukey's post hoc test significances indicated in the graph; Fig. 1F]. Interestingly, *mao*^+/−^ larvae showed a significantly increased startle response compared with their siblings on the second, third and fourth stimuli. *mao*^−/−^ larvae habituated faster to repeated exposure of the acoustic/vibrational stimuli at 20 s interstimulus interval (ISI), whereas their siblings did less so.

**Fig. 2.**
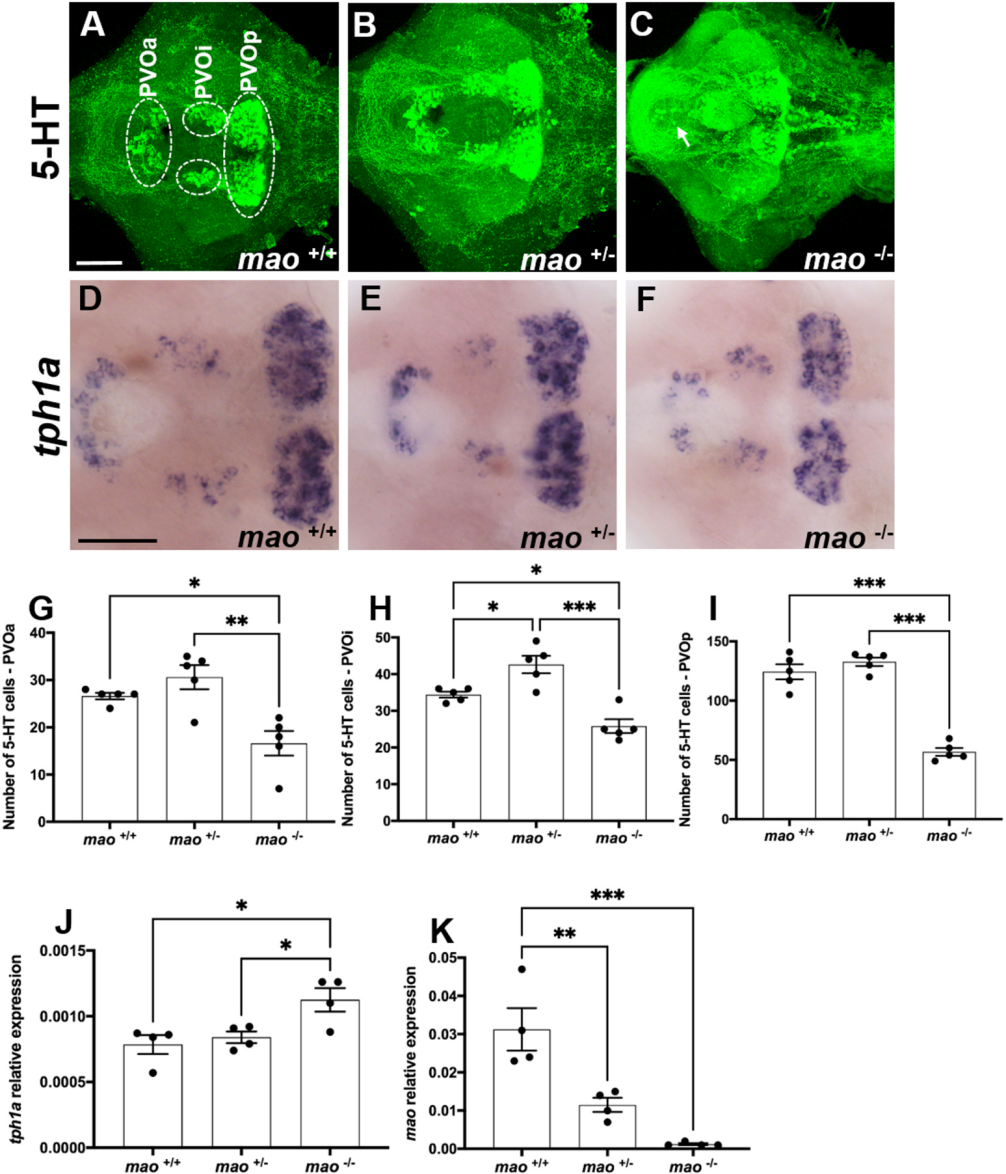
**The serotonergic system of *mao* mutants.** (A-C) Ventral views of whole-mount 10 dpf *mao*^+/+^ (A), *mao*^+/−^ (B) and *mao*^−/−^ (C) larval brains, anterior to the left, processed for serotonin (5-HT) immunostaining. Arrow indicates ectopic cells in *mao*^−/−^ brain. *n*=5 for each genotype. (D-F) Ventral views of whole-mount 10 dpf *mao*^+/+^ (D), *mao*^+/−^ (E) and *mao*^−/−^ (F) larval brains, anterior to the left, processed for *tph1a* RNA *in situ* hybridization (ISH). (G-I) Bar charts showing results of 5-HT-immunoreactive cell counting in larval brains [paraventricular organ anterior part (PVOa; G), paraventricular organ intermediate part (PVOi; H) and paraventricular organ posterior part (PVOp; I)] of the indicated genotype at 10 dpf. (J,K) Bar charts showing results of reverse transcriptase-quantitative PCR (RT-qPCR) analysis of *tph1a* (J) and *mao* (K) in larvae of the indicated genotype at 10 dpf. Data are mean±s.e.m. One-way ANOVA followed by Tukey's post hoc test was used for statistical analysis. *P<0.05, **P<0.01, ***P<0.001. Scale bars: 75 μm.

Both *mao*^+/−^ and *mao*^−/−^ larvae presented stronger 5-HT immunoreactivity than that of their *mao*^+/+^ siblings at 10 dpf (*n*=5 for each genotype; Fig. 2A-C), which is likely to reflect increased levels of this neurotransmitter, a phenomenon already described in zebrafish larvae treated with a Mao inhibitor (Sallinen et al., 2009b). However, *mao*^−/−^ larvae presented a reduced number of 5-HT-positive cells in the paraventricular organ anterior part [PVOa; *n*=5 for each genotype; *F*(2, 12)=11.40, *P*<0.01; Fig. 2G], paraventricular organ intermediate part [PVOi; *n*=5 for each genotype; *F*(2, 12)=21.47, *P*<0.01; Fig. 2H] and paraventricular organ posterior part [PVOp; *n*=5 for each genotype; *F*(2, 12)=81.84, *P*<0.01; Fig. 2I] compared with both *mao*^+/+^ and *mao*^−/−^ siblings. This indicates that the stronger immunoreactivity is against extracellular 5-HT and fibers. They also presented ectopic 5-HT-immunoreactive cells in the preoptic area, which are unlikely to be 5-HT-synthesizing cells, but rather cells that have taken up 5-HT, which is found in extracellular space (Fig. 2C). *mao*^+/−^ larvae presented a higher number of 5-HT-positive cells in the PVOi compared with their *mao*^+/+^ and *mao*^−/−^ siblings, but no differences between *mao*^+/+^ and *mao*^+/−^ larvae were detected in the PVOa and PVOp (Fig. 2G-I).

A weaker *tph1a* signal was detected in the brains of *mao*^−/−^ larvae compared with that in the brains of their *mao*^+/+^ and *mao*^+/−^ siblings (*n*=5 for each genotype; Fig. 2D-F). Interestingly, when we quantified *tph1a* mRNA by quantitative PCR (qPCR), *mao*^−/−^ larvae at 10 dpf presented significantly higher expression of this transcript compared with their *mao*^+/+^ and *mao*^+/−^ siblings [*n*=4 for each genotype; *F*(2, 9)=6.552, *P*<0.05; Fig. 2J]. Zebrafish *mao*^+/−^ and *mao*^−/−^ larvae had a significant reduction in *mao* mRNA compared with their *mao*^+/+^ siblings at 10 dpf [*n*=5 for each genotype; *F*(2, 9)=20.39, *P*<0.01; Fig. 2K]. *mao*^−/−^ larvae died within 20 days after birth, whereas *mao*^+/−^ larvae were viable and survived to adulthood.

### Dopaminergic and histaminergic alterations in *mao*^−/−^ larvae

**Fig. 3.**
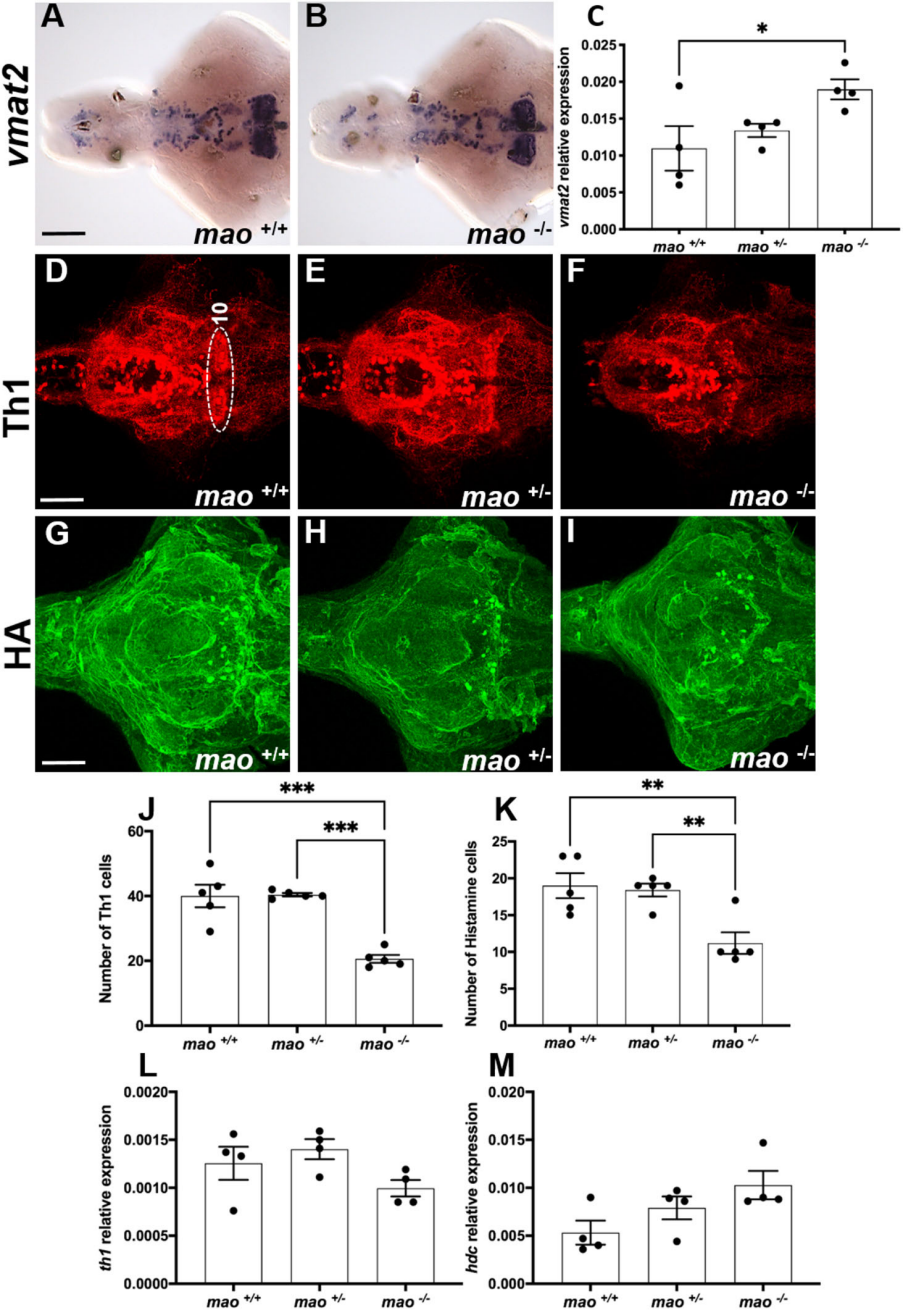
**The dopaminergic and histaminergic systems of *mao* mutants.** (A,B) Ventral views of whole-mount 10 dpf *mao*^+/+^ (A) and *mao*^−/−^ (B) larval brains, anterior to the left, processed for *vmat2* RNA ISH. *n*=4 for each genotype. (C) Bar charts showing results from RT-qPCR analysis of *vmat2* in larvae of the indicated genotype at 10 dpf. *n*=4 for each genotype. (D-F) Ventral views of whole-mount 10 dpf *mao*^+/+^ (D), *mao*^+/−^ (E) and *mao*^−/−^ (F) larval brains, anterior to the left, processed for tyrosine hydroxylase 1 (Th1) immunostaining. *n*=5 for each genotype. (G-I) Ventral views of whole-mount 10 dpf *mao*^+/+^ (G), *mao*^+/−^ (H) and *mao*^−/−^ (I) larval brains, anterior to the left, processed for histamine (HA) immunostaining. *n*=5 for each genotype. (J,K) Bar charts showing results from Th1- and histamine-immunoreactive cell counting in larval brains of the indicated genotype at 10 dpf. (L,M) Bar charts showing results from RT-qPCR analysis of *th1* (L) and *hdc* (M) in larvae of the indicated genotype at 10 dpf. Data are mean±s.e.m. One-way ANOVA followed by Tukey's post hoc test was used for statistical analysis. *P<0.05, **P<0.01, ***P<0.001. Scale bars: 75 μm.

No evident differences in the expression pattern of *vesicular monoamine transporter 2* (*vmat2*; also known as *slc18a2*), encoding a transmembrane protein and key regulator of the monoaminergic systems by transporting cytoplasmic monoamines into presynaptic vesicles (Baronio et al., 2021), were observed with *in situ* hybridization (ISH) between *mao*^−/−^ and *mao*^+/+^ larval brains (*n*=4 for each genotype; Fig. 3A,B). However, the reverse transcriptase-qPCR (RT-qPCR) analysis revealed that *vmat2* mRNA was upregulated in whole *mao*^−/−^ larvae compared with *mao*^+/+^ larvae [*n*=4 for each genotype; *F*(2, 9)=4.286, *P*<0.05; Fig. 3C]. When we evaluated the dopaminergic and histaminergic systems of *mao*^+/+^, *mao*^+/−^ and *mao*^−/−^ larvae by immunohistochemistry at 10 dpf, we detected a reduction in the number of tyrosine hydroxylase 1 (Th1)-immunoreactive cells in cell group 10 of the posterior hypothalamus [*n*=5 for each genotype; *F*(2, 12)=28.01, *P*<0.01; Fig. 3D-F,J] and histamine-immunoreactive cells [*n*=5 for each genotype; *F*(2,12)=9.745, *P*<0.01; Fig. 3G-I,K] in *mao*^−/−^ brains compared with the brains of their *mao*^+/+^ and *mao*^+/−^ siblings. However, no overall difference between genotypes was seen when the *th1* (the first and rate-limiting step in synthesis of DA and other catecholamines) transcript levels of *mao*^+/+^, *mao*^+/−^ and *mao*^−/−^ larvae were analyzed in whole larvae at 10 dpf [*n*=4 for each genotype; *F*(2, 9)=2.659, *P*>0.05; Fig. 3L]. Similarly, the levels of *hdc* (encoding histidine decarboxylase, the enzyme responsible for catalyzing the conversion of histidine to histamine) mRNA were not statistically different when the three genotypes were compared [*n*=4 for each genotype; *F*(2, 9)=3.571, *P*>0.05; Fig. 3M].

### Impaired neuronal development of *mao*^−/−^ larvae

**Fig. 4.**
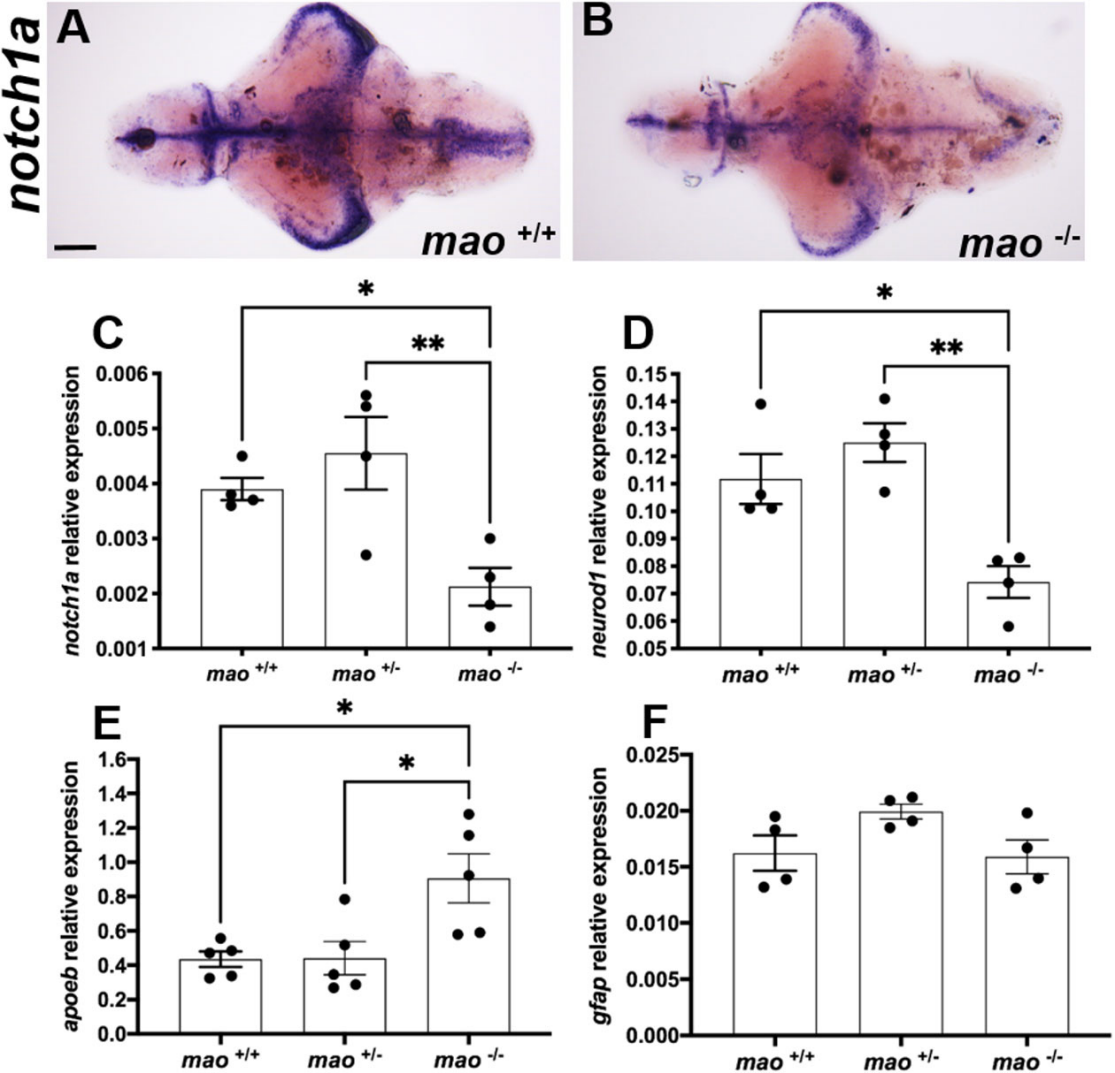
***mao*^−/−^ larvae exhibit altered expression of developmental markers.** (A,B) Ventral views of whole-mount 10 dpf *mao*^+/+^ (A) and *mao*^−/−^ (B) larval brains, anterior to the left, processed for *notch1a* RNA ISH. *n*=4 for each genotype. (C-F) Bar charts showing results from RT-qPCR analysis of *notch1a* (C), *neurod1* (D), *apoeb* (E) and *gfap* (F) in larvae of the indicated genotype at 10 dpf. *n*=4 for each genotype. Data are mean±s.e.m. One-way ANOVA followed by Tukey's multiple comparisons test for statistical analysis. *P<0.05, **P<0.01. Scale bar: 75 μm.

*mao*^−/−^ larvae showed a weaker signal for the early neuronal marker *notch1a* mRNA after ISH compared with their *mao*^+/+^ siblings (*n*=4 for each genotype; Fig. 4A,B). This result was confirmed by RT-qPCR, which demonstrated a significant reduction in *notch1a* mRNA compared with their *mao*^+/+^ and *mao*^+/−^ siblings [*n*=4 for each genotype; *F*(2,9)=7.784, *P*<0.05; Fig. 4C]. *mao*^−/−^ larvae also presented downregulation of *neurod1* mRNA [*n*=4 for each genotype; *F*(2,9)=12.49, *P*<0.01; Fig. 4D], encoding a transcription factor that is critical for survival and maturation of newborn neurons (Gao et al., 2009), compared with both *mao*^+/+^ and *mao*^+/−^ siblings. A significant increase in *apolipoprotein Eb* (*apoeb*) mRNA [*n*=4 for each genotype; *F*(2,9)=7.039, *P*<0.05; Fig. 4E], encoding a microglial marker (Elliott et al., 2007), was detected in *mao*^−/−^ larvae compared with both *mao*^+/+^ and *mao*^+/−^ siblings. No difference between the genotypes was detected when the levels of glial marker *gfap* mRNA were evaluated [*n*=4 for each genotype; *F*(2, 9)=2.895, *P*>0.05; Fig. 4F].

### *mao^+/−^* adult fish display abnormal social interaction and weaker Mao activity

**Fig. 5.**
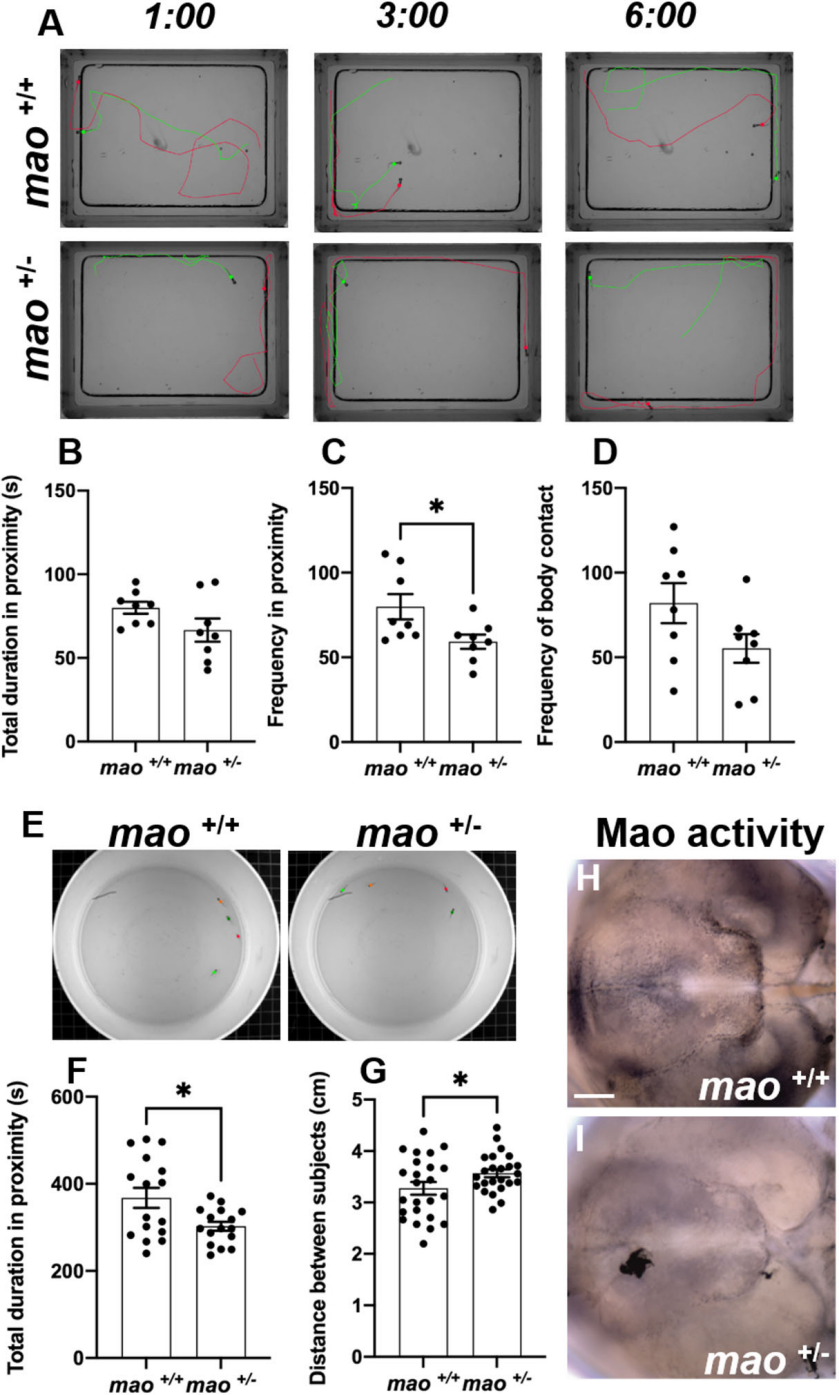
**Social cohesion of *mao*^+/−^ juvenile fish is impaired.** (A) Representative images of three different time points during the social contact behavior of *mao*^+/+^ and *mao*^+/−^ fish at 30 dpf. *n*=8 for each genotype. (B,C) Bar charts showing total duration (B) and frequency (C) in proximity to other fish for the indicated genotypes. (D) Bar charts showing frequency of body contact for the indicated genotypes. (E) Representative images of 40 dpf *mao*^+/+^ and *mao*^+/−^ fish during the shoaling behavior evaluation. *n*=4 for each genotype. (F) Bar charts showing total duration in proximity to other fish during shoaling behavior for the indicated genotypes. (G) Bar charts showing average distance between fish of the indicated genotype during shoaling behavior. (H,I) Ventral views of whole-mount 40 dpf *mao*^+/+^ (H) and *mao*^−/−^ (I) brains, anterior to the left, processed for Mao activity. *mao*^+/−^ fish show weaker Mao activity after shoaling behavior. *n*=4 for each genotype. Data are mean±s.e.m. Unpaired, two-tailed Student's *t*-test was used for statistical analysis. *P<0.05. Scale bar: 75 μm.

Juvenile *mao*^+/+^ and *mao*^+/−^ fish (30 dpf) were evaluated in a social contact assay in which a pair of fish of the same genotype were placed in an arena for 6 min (Fig. 5A). The total duration in proximity (at a distance ≤0.8 cm) was not different when *mao*^+/+^ and *mao*^+/−^ fish were compared (*n*=8 pairs for each genotype; *P*>0.05; Fig. 5B). However, *mao*^+/−^ fish were less frequently in proximity to each other (*n*=8 pairs for each genotype; *P*<0.05; Fig. 5C). Additionally, we evaluated the frequency of direct contact between the fishes in the arena, but no statistically significant difference was detected when the genotypes were compared (*n*=8 pairs for each genotype; *P*>0.05; Fig. 5D).

To test whether *mao* deficiency affects fish shoaling behavior, four 40 dpf fish per trial were placed in a cylindrical plastic container (Fig. 5E). The average distance between the test fish and the other three shoal members and total duration in proximity to the other fish (the nearest interindividual distance defined as 1 cm) were analyzed. The time spent in proximity to shoal members was significantly shorter in the *mao*^+/−^ fish group than in their *mao*^+/+^ siblings (*n*=4 trials for each genotype; *P*<0.05; Fig. 5F). Furthermore, the interindividual distance was significantly greater in the *mao*^+/−^ group than in the *mao*^+/+^ sibling group (*n*=4 trials for each genotype; *P*<0.05; Fig. 5G). After the trial, the brains of fish from both genotypes were dissected, and the Mao activity histochemical assay was performed (*n*=4 for each genotype; Fig. 5H,I). *mao*^+/−^ brains showed weaker Mao activity in the hypothalamus area compared with the *mao*^+/+^ sibling brains.

**Fig. 6.**
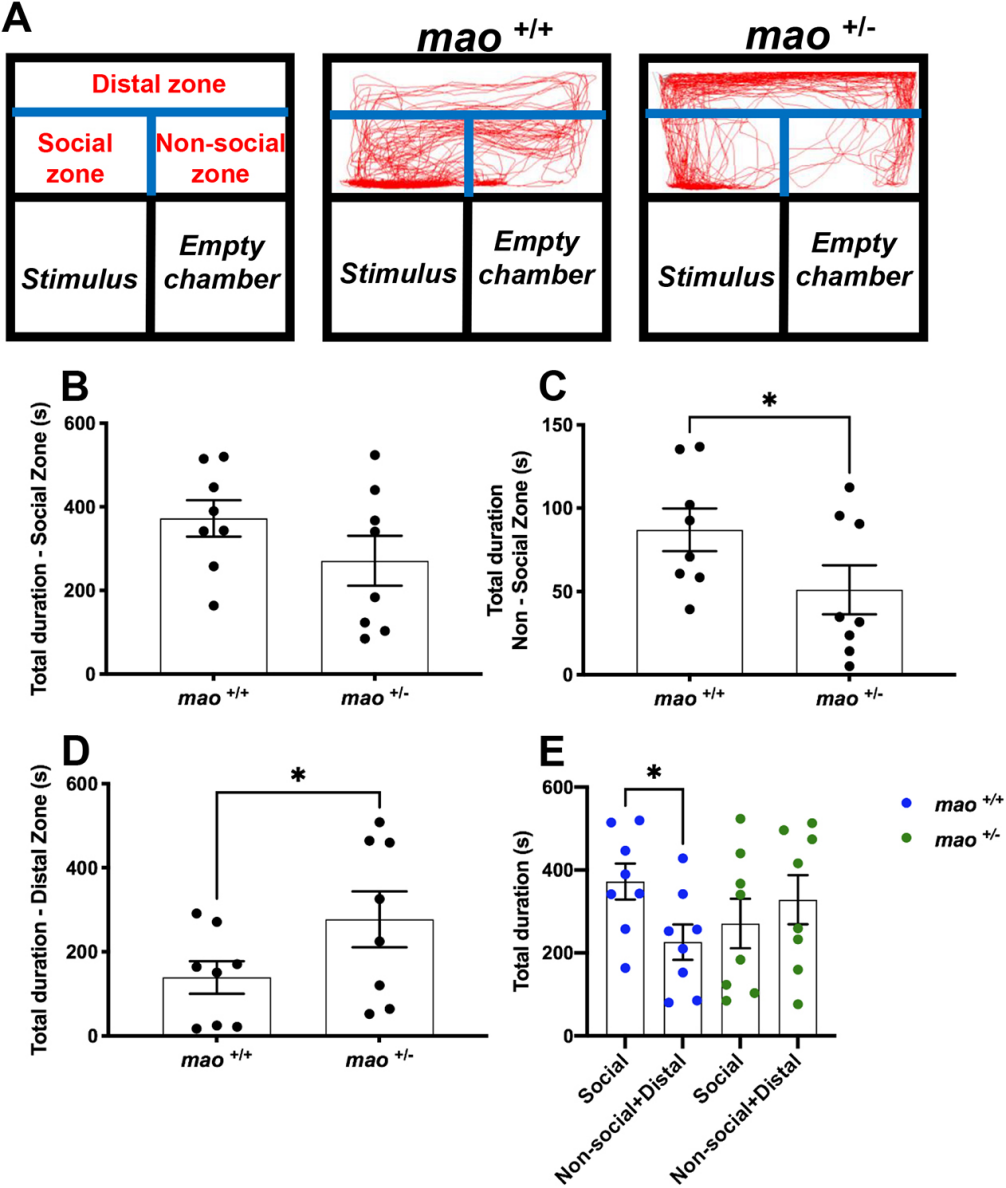
**Social preference of *mao*^+/−^ adult fish is impaired.** (A) Representation of the apparatus used to study the social behavior of adult *mao*^+/+^ and *mao*^+/−^ fish and traces of the swimming pattern from both genotypes. Fish behavior was evaluated during a 10 min video recording session in a home-made social interaction apparatus. The apparatus is physically divided into three chambers and digital zones (distal, social and non-social), which were established with EthoVision XT 13 software. *n*=8 for each genotype. (B-E) Bar charts showing results on the time spent in each zone [social (B), non-social (C), distal (D) and total (E)]. Compared to their siblings, *mao*^+/−^ adult fish did not differ in the time spent closer to the social stimulus, spent less time in the non-social zone and more time in the distal zone. When the time spent in the distal zone and non-social zones was summed, the *mao*^+/+^ fish show a preference for the area closer to the social stimulus, whereas when the same comparison is done with their *mao*^+/−^ siblings, no preference is detected. Data are mean±s.e.m. Unpaired, two-tailed Student's *t*-test was used for statistical analysis. *P<0.05.

Finally, the social preference of adult fish was tested. Fig. 6A shows the traces of the swimming pattern of *mao*^+/+^ and *mao*^+/−^ adult fish during the social interaction test. Compared with their *mao*^+/+^ siblings, *mao*^+/−^ adult fish did not differ statistically in the time spent in the zone closest to the compartment with the stimulus fish (*n*=8 for each genotype; *P*>0.05; Fig. 6B) and spent less time in the non-social zone (*n*=8 for each genotype; *P*<0.05; Fig. 6C). However, *mao*^+/−^ adult fish preferred to stay longer in the distal area of the apparatus (*n*=8 for each genotype; *P*<0.05; Fig. 6D). When we compared the time spent in the social zone by the *mao*^+/+^ fish with the time spent in the distal zone and non-social zone summed, these animals showed a preference for the area closer to the social stimulus detected (*n*=8 for each genotype; *P*<0.05; Fig. 6E), whereas when the same comparison was made with their *mao*^+/−^ siblings, no significant difference was detected.

### *mao^+/+^* and *mao^+/−^* adult fish display similar locomotor activity and thigmotaxis

**Fig. 7.**
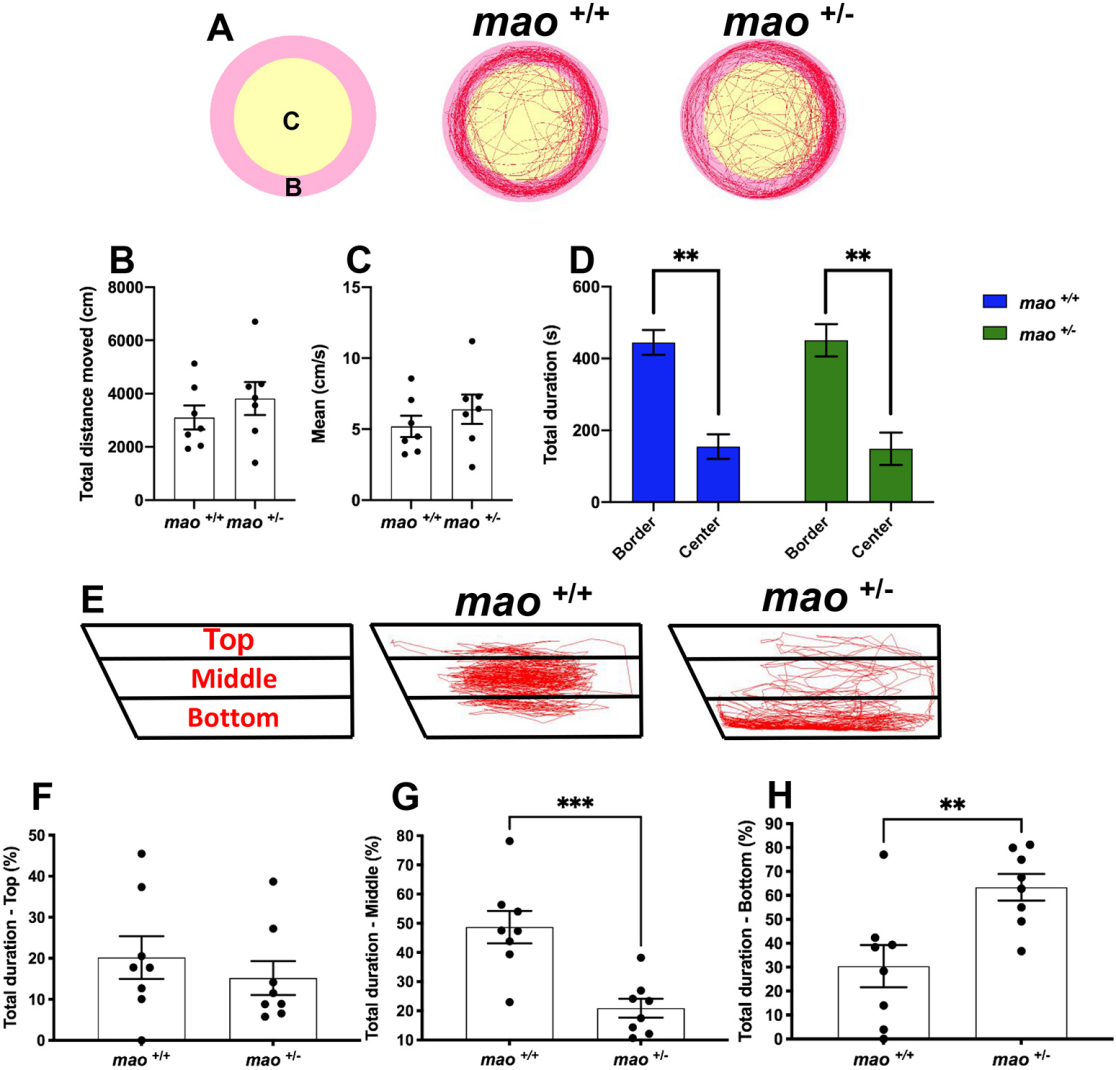
**Locomotor activity and novel tank test of adult *mao*^+/+^ and *mao*^+/−^ fish.** (A) Representation of the arena with digital zones and representative swimming traces of *mao*^+/+^ and *mao*^+/−^ fish during a 10 min recording period. *n*=7 for each genotype. (B,C) Bar charts showing individual values for total distance moved (B) and average velocity (C) of fish of the indicated genotype. (D) Bar charts showing thigmotaxis larval behavior. (E) Schemes of the novel tank test with three digital zones and representative swimming traces of *mao*^+/+^ and *mao*^+/−^ fish during a 6 min recording period. *n*=8 for each genotype. (F-H) Bar charts showing results on the time spent in each zone [top (F), middle (G) and bottom (H)]. Compared to their siblings, *mao*^+/−^ adult fish did not differ in the time spent in the upper part of the tank, spent less time in the middle and more time in the bottom part. Data are mean±s.e.m. Unpaired, two-tailed Student's *t*-test was used for statistical analysis. **P<0.01, ***P<0.001.

Schemes of the locomotor activity test with two digitized zones and representative movement traces are shown in Fig. 7A. Locomotor activity, measured as total distance moved, did not significantly differ between *mao*^+/+^ and *mao*^+/−^ adult fish during a 10 min trial (*n*=7 for each genotype; *P*>0.05; Fig. 7B). No differences in velocity were detected (*n*=7 for each genotype; *P*>0.05; Fig. 7C). During the same trial, we also assessed thigmotaxis, a behavior in which animals spend most of the time of a session near the walls of the apparatus (Maximino et al., 2010). No difference between the genotypes was detected in this behavior, with both *mao*^+/+^ and *mao*^+/−^ adult fish spending most of the trial in the zone close to the arena wall (*n*=7 for each genotype; *P*<0.01; Fig. 7D).

### *mao^+/−^* adult fish display anxiety-like behavior in the novel tank diving test

The initial preference of the zebrafish for the bottom of a novel tank, and subsequent exploration of the rest of the tank, is commonly interpreted as a precautionary antipredator response followed by alleviation of anxiety, respectively. We used a novel tank diving assay to study anxiety-related risk-taking behavior of *mao*^+/+^ and *mao*^+/−^ adult fish. The novel tank diving area was digitally divided into three zones, and representative swimming tracks are shown in Fig. 7E. Compared with their *mao*^+/+^ siblings, *mao*^+/−^ adult fish spent similar time exploring the top zone (*n*=8 for each genotype; *P*>0.05; Fig. 7F). However, they spent less time in the middle zone (*n*=8 for each genotype; *P*<0.05; Fig. 7G) and longer time in the bottom zone of the tank (*n*=8 for each genotype; *P*<0.05; Fig. 7H).

### Gene expression in *mao*^+/−^ adult zebrafish brains

**Fig. 8.**
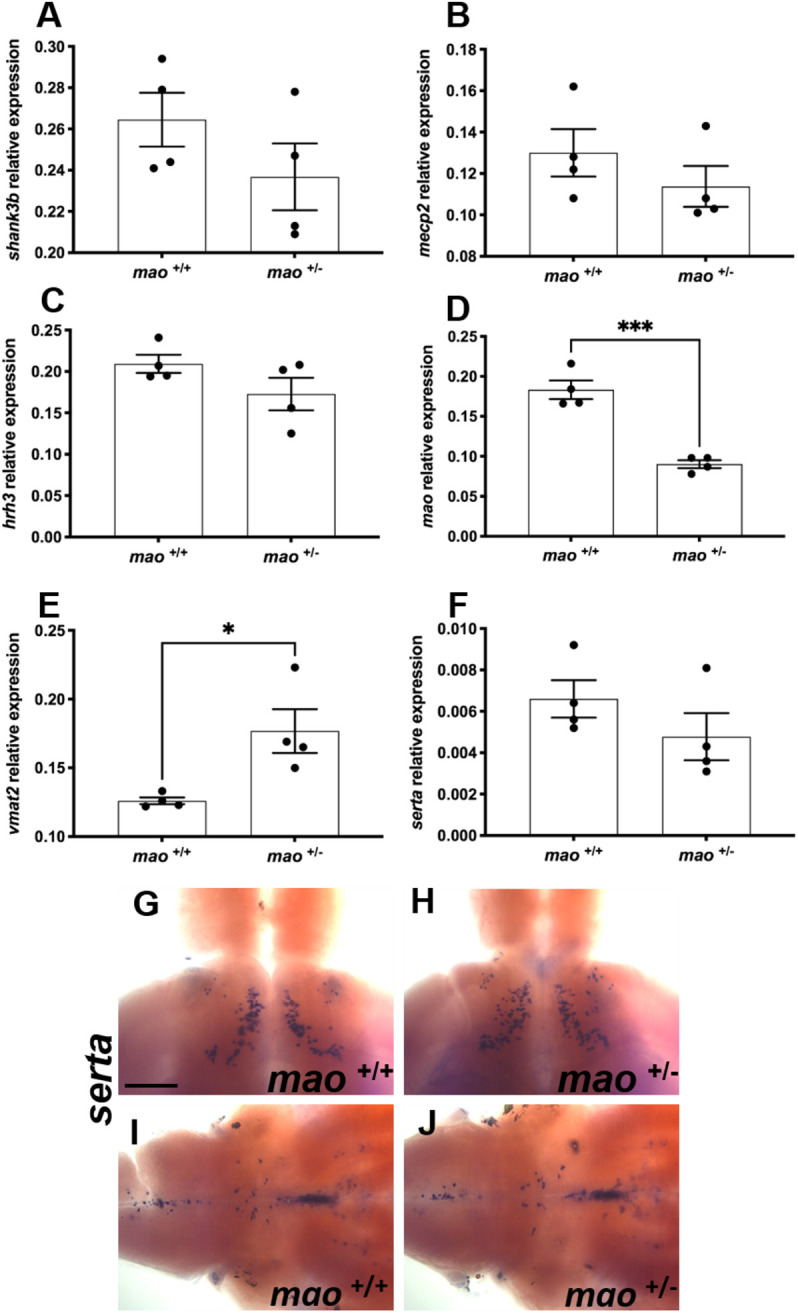
**Gene expression in adult *mao*^+/+^ and *mao*^+/−^ fish.** (A-F) Bar charts showing results from RT-qPCR analysis of *shank3b* (A), *mecp2* (B), *hrh3* (C), *mao* (D), *vmat2* (E) and *serta* (F) in adult brains of the indicated genotype. A significant decrease in the expression of *mao* was detected in *mao*^+/−^ fish compared to *mao*^+/+^ siblings. A significant upregulation in the expression of *vmat2* was detected in *mao*^+/−^ fish compared to *mao*^+/+^ siblings. (G,H) Dorsal views of whole-mount adult *mao*^+/+^ (G) and *mao*^+/−^ (H) brains, anterior to the top, processed for *serta* RNA ISH. *serta*-positive neurons expressed in the ventral posterior tuberculum. (I,J) Ventral views of whole-mount adult *mao*^+/+^ (I) and *mao*^+/−^ (J) brains, anterior to the right, processed for *serta* RNA ISH. *serta*-positive neurons expressed in the raphe nuclei and hindbrain. Data are mean±s.e.m. Unpaired, two-tailed Student's *t*-test was used for statistical analysis. *P<0.05, ***P<0.001. Scale bar: 75 μm.

We investigated the brains of *mao*^+/−^ adult fish to detect possible alterations that could be associated with their impaired behavior. *SH3 and multiple ankyrin repeat domains 3* (*shank3b*) and *methyl CpG binding protein 2* (*mecp2*) transcripts were quantified because of the extensive literature associating these genes with ASD (Muhle et al., 2004; Pardo and Eberhart, 2007), but no differences were detected when the genotypes were compared (*n*=4 for each genotype; *P*>0.05; Fig. 8A,B). *histamine receptor h3* (*hrh3*) plays roles in anxiety and cognition, and recent findings suggest that this receptor could be a potential therapeutic target for ASD (Baronio et al., 2015; Eissa et al., 2018), but *mao*^+/+^ and *mao*^+/−^ brains presented similar levels of this transcript (*n*=4 for each genotype; *P*>0.05; Fig. 8C). As expected, the *mao*^+/−^ brains displayed a significantly reduced level of *mao* mRNA compared with the brains of *mao*^+/+^ siblings (*n*=4 for each genotype; *P*<0.01; Fig. 8D). We quantified *vmat2* transcript levels to assess this key factor in monoaminergic neurotransmission (Fon et al., 1997). *vmat2* mRNA was upregulated in *mao*^+/−^ fish brains (*n*=4 for each genotype; *P*<0.05; Fig. 8E). In zebrafish, *serotonin transporter a* (*serta*; also known as *slc6a4a*)-positive neurons are found in the raphe nuclei and the ventral posterior tuberculum. A similar distribution pattern of *serta* was detected in both *mao*^+/+^ and *mao*^+/−^ zebrafish (*n*=4 for each genotype; *P*>0.05; Fig. 8G-J). qPCR analysis using whole brains as samples indicated equal expression levels of *serta* mRNA in *mao*^+/+^ and *mao*^+/−^ zebrafish (*n*=4 for each genotype; *P*>0.05; Fig. 8F).

## DISCUSSION

*mao* loss of function in *mao*^−/−^ zebrafish larvae led to hypoactivity and abnormal serotonergic, dopaminergic and histaminergic systems. The expression of important developmental markers was altered, and *mao*^−/−^ larvae died within 20 dpf. *mao*^+/−^ animals were viable, grew until adulthood, and demonstrated anxiety-like behavior and impaired social interaction.

The hypoactive phenotype was detected during a 24 h basic locomotor activity evaluation in which the distance moved by *mao*^−/−^ larvae was significantly decreased compared with that moved by *mao*^+/+^ and *mao*^+/−^ siblings. This is in agreement with a previous report in which Mao inhibition in larval wild-type (WT) zebrafish decreases locomotion (Sallinen et al., 2009b). Additionally, weaker reactivity to acoustic/vibrational and visual stimuli was displayed by *mao*^−/−^ larvae. This could be a consequence of the severe hypoactive phenotype and general arrested development displayed by the mutants, considering that these responses are mediated by independent processes. The Mauthner cells modulate the escape response in zebrafish after acoustic/vibrational stimuli. After such stimuli, larvae will display a short latency response with an increase in locomotor activity and velocity. This phenotype is thought to be a form of predation avoidance. A hyperactive phenotype is also noted when there is abrupt change in illumination, such as in the dark-flash response test. Initially, it was hypothesized that this was also an escape response to predation. However, it has been shown that this behavior is not mediated by the Mauthner cells and is more likely to be navigational rather than defensive (Burgess and Granato, 2007). Interestingly, *mao*^+/−^ larvae showed difficulty to adapt to repeated acoustic/vibrational stimuli in comparison to *mao*^+/+^ and *mao*^−/−^ larvae. The startle response is associated with cognitive processing of sensory information, which is important for modeling cognitive deficits, resembling those associated with anxiety (Pittman and Lott, 2014). A similar result was obtained when dark-flash response was evaluated, and *mao*^+/−^ larvae displayed a significantly stronger reaction than *mao*^+/+^ and *mao*^−/−^ larvae in the first dark flash. Additionally, compared with *mao*^+/+^ larvae, *mao*^+/−^ larvae took longer to adapt to the change in illumination and to return to the baseline activity.

Mao inhibition in larval WT zebrafish leads to increased levels of 5-HT (Sallinen et al., 2009b). When we quantified 5-HT-positive neurons in different serotonergic groups of the brain in *mao*^−/−^ larvae, we verified a reduced number of 5-HT-immunoreactive cells. However, strong extracellular 5-HT immunoreactivity, as well as increased density of immunoreactive fibers, could be noted. It is possible that the excess of 5-HT led to a toxic effect, causing cell death. Similar phenotype was reported when the brains of larval WT zebrafish treated with a Mao inhibitor presented reduced 5-HT immunoreactivity in the serotonergic cell somata, extracellular 5-HT immunoreactivity and increased density of 5-HT-immunoreactive fibers (Sallinen et al., 2009b).

ISH showed a weaker *tph1a* signal in larval *mao*^−/−^ brains than in *mao*^+/+^ and *mao*^+/−^ brains. However, a significantly higher expression of this transcript was detected by RT-qPCR in whole *mao*^−/−^ larvae compared with *mao*^+/+^ and *mao*^+/−^ siblings. One explanation for the discrepancy between these results is that, for the RT-qPCR analysis, pools of whole larvae were used as samples, and *tph1a* is expressed in skin and pharyngeal arch neuroepithelial cells (NECs) and nerves innervating NECs (Pan et al., 2021).

The likely neurotoxicity caused by the excess of 5-HT might have affected other monoaminergic populations and contributed to the early death of the *mao*^−/−^ mutant, as we detected reduced numbers of histaminergic and Th1-immmunoreactive neurons in the brains of *mao*^−/−^ larvae, compared with the brains of their *mao*^+/+^ and *mao*^+/−^ siblings. Furthermore, the microglial marker *apoeb* was upregulated in *mao*^−/−^ larvae, raising the possibility of increased apoptosis in these larvae (Elliott et al., 2007). It has been reported that zebrafish exposed to trans-2-phenylcyclopropylamine (PCPA), a non-selective MAO inhibitor, present increased neuronal death (Jie et al., 2009). Contributions of these hypothalamic systems to behaviors in larval zebrafish have been described, including regulation of feeding (Wee et al., 2019). Additionally, zebrafish swim bladder has been reported to receive serotonergic input (Finney et al., 2006), and Mao inhibition is known to affect swim bladder control in zebrafish (Sallinen et al., 2009b), which could have compromised the ability of the larvae to move and seek for food. Thus, decreased food intake could have also contributed to their death.

An inherited 240 kb deletion on Xp11.3-p11.4, which encompasses both *MAOA* and *MAOB* genes, has been reported in two human male siblings (Whibley et al., 2010). Both brothers had severe mental retardation, hand wringing, lip smacking and epilepsy. One of them died at the age of 5, and the autopsy did not reveal any abnormalities of his internal organs except for his brain, which was mildly underweight and showed loss of Purkinje cells in the cerebellum and some loss of neurons in the cortex. Zebrafish possess only one *mao*, and the gene has similarity in exon/intron numbers and structure to *MAOA* and *MAOB* of humans, while the protein shares the properties of both human MAOA and MAOB. Additionally, the overall distribution of zebrafish *mao* is a combination of *Maoa* and *Maob* distribution in mammals (Anichtchik et al., 2006). Aminergic systems are highly conserved in zebrafish brain, and the transparency of the zebrafish embryo makes it possible to rapidly assess the effects of small molecules on many aspects of anatomy or physiology (Kaslin and Panula, 2001; Panula et al., 2010). Thus, *mao*^−/−^ larvae could be an alternative tool to study the consequences of MAO loss of function during early development and potential therapeutic approaches that could prevent or restore a hyperserotonergic phenotype.

Based on human and rodent evidence suggesting an association between *MAOA* and ASD (Bortolato et al., 2011; Cohen et al., 2003; Gu et al., 2017), we aimed to verify whether *mao*^+/−^ adult zebrafish show autistic-like social deficits. These animals presented decreased Mao activity, a marked reduction in *mao* mRNA, stronger 5-HT immunoreactivity and an increased number of 5-HT-positive cells in the PVOi in comparison with their *mao*^+/+^ siblings.

Signs of mild impairment in social behavior in juvenile *mao*^+/−^ fish were detected at 30 dpf, when, in a paradigm that evaluates social contact, they were less frequently in proximity to another fish of same genotype. When shoaling behavior was evaluated at 40 dpf, we verified that juvenile *mao*^+/−^ fish in groups of four individuals spent less time in proximity, and that there was an increased average distance between subjects compared with the trials containing *mao*^+/+^ siblings. Shoaling behavior is an innate form of social interaction displayed by zebrafish that serves different purposes in their natural habitat (Pitcher, 1986). Additionally, *mao*^+/−^ adult zebrafish did not show a preference for a social stimulus when evaluated in a social interaction apparatus. These behavioral alterations could be a consequence of yet unknown developmental defects caused by Mao deficiency in *mao*^+/−^ larvae, rather than a direct effect of Mao deficiency in juvenile and adult fish.

Overall, *mao*^+/−^ zebrafish presented only a mild impairment in social behavior. This is a caveat that must be considered when using this research tool to model brain disorders for which impaired sociability is one of the core symptoms. However, because environmental and genetic factors are known to have a role in the pathophysiology of neurodevelopmental disorders, *mao*^+/−^ fish could be considered in future studies that aim to investigate whether specific gene–environment interactions can shape the vulnerability for distinct neuronal and neurocognitive abnormalities relevant for multifactorial and monoaminergic-associated neurodevelopmental disorders. For instance, it was suggested that children who suffered early trauma and have the *MAOA* genotype that confers low levels of MAOA enzyme are more likely to develop antisocial behavior in adulthood than maltreated children with a high-activity *MAOA* genotype (Piton et al., 2014).

In comparison with their *mao*^+/+^ siblings, *mao*^+/−^ adult zebrafish spent longer time in the bottom of the novel tank when the diving test was performed. This phenomenon may be related to the natural instinct of zebrafish to initially seek protection in an unfamiliar environment, avoiding the surface, and it is commonly used as an index of anxiety-like responsiveness. *Maoa* KO mice and *Maob* KO mouse behavior do not significantly differ from that of WT mice in the elevated plus maze, indicating that they do not display anxious-like behavior (Cases et al., 1995; Grimsby et al., 1997). However, MAOA/B KO mice display locomotor inhibition and avoidance of the center of the arena in the open-field test, and smaller number of entries into both the open and closed arms of the plus maze (Chen et al., 2004). MAOA hypomorphic mutants also showed a reduction in total locomotor activity in a novel open field and significant preference to spend less time in the central zone of the arena in the open field. On the other hand, MAOA hypomorphic mice do not show anxiety-like responses in the elevated plus maze and the light–dark box (Bortolato et al., 2011).

We evaluated the expression of relevant genes in the brains of adult *mao*^+/−^ fish and *mao*^+/+^ siblings and found a significant increase in expression of *vmat2*, encoding a vesicular transporter essential for monoamine release (Fon et al., 1997), in *mao*^+/−^ brains. An increase was also detected in *mao*^−/−^ larval samples. The decreased *mao* expression and activity is expected to increase the levels of 5-HT in zebrafish (Sallinen et al., 2009b). Thus, a compensatory upregulation of *vmat2* would not be surprising. In fact, rats treated with MAO inhibitor tranylcypromine from gestational day 12 until birth through subcutaneous injections to pregnant females, and from postnatal day 1 until postnatal day 21 through subcutaneous injections, presented increased *Vmat2* mRNA levels in the raphe nuclei region when they reached adulthood (Blažević and Hranilović, 2013). Interestingly, radioligand binding and autoradiography assays have demonstrated that the numbers of VMAT2 sites are modestly decreased in MAOA KO mice (Shih et al., 1999). The discrepancy between our result and the ones obtained utilizing MAOA KO mice could be attributed to the different methods and model organisms utilized in the two studies. Increased VMAT2 concentration has been detected in the brains of patients with schizophrenia (Zubieta et al., 2001), a disorder that has overlaps in symptoms with ASD (Trevisan et al., 2020).

Abnormalities in the serotonergic system have been linked with ASD, reports of hyperserotonemia (Veenstra-VanderWeele et al., 2012), decreased MAO activity (Gu et al., 2017; Young et al., 1980) and different reports regarding the serotonin transporter (SERT; also known as SLC6A4). Increased SERT expression in ASD has been associated with stereotypies (Mulder et al., 2005). In contrast, decreased SERT expression has been linked to impaired social behavior (Brune et al., 2006). However, in a positron emission tomography study of SERT binding in adults with Asperger's disorder, no alterations were found (Girgis et al., 2011). Here, we report no difference in the expression of *serta* in the brains of *mao*^+/−^ fish in comparison with the brains of their *mao*^+/+^ siblings after whole-brain qPCR analysis.

In summary, the present work contributes to previous pharmacological data concerning the behavioral and neurochemical consequences of *mao* inactivation in zebrafish. The monoaminergic systems regulate neuronal growth, differentiation, migration and survival. Thus, disrupted monoaminergic systems can lead to impairments in brain function and mental illness, making *mao*^−/−^ fish a promising tool to study the roles of *MAOA/B* and monoamines during brain development. *mao*^+/−^ fish present mild impairment in social behavior and anxiety, and thus are a potential tool for studies that aim to assess the developmental and behavioral outcomes of interaction between environmental factors and *MAOA/B* genotype.

## MATERIALS AND METHODS

### *mao* mutant

The *mao* mutant (sa^31732^) was generated by the Sanger Institute Zebrafish Mutation Project and contains an A/T nonsense mutation at nucleotide 685 of the open-reading frame, which is predicted to generate a 229-amino acid protein compared with the 522-amino acid WT protein.

Larvae were raised on 14:10 h (light/dark, lights on at 08:00) cycles at 28°C and fed once with flake food and two times with live artemia daily. Adult fish were raised in a continuously cycling Aquatic Habitats Systems with complete exchange of water in each tank every 6-10 min. Circulating water was UV sterilized, and filtered with foam filters and activated charcoal. Water quality, including temperature (28±0.5°C), pH value (7.4±0.2) and conductivity (450±10 mS), was monitored continuously. Embryos were obtained by natural spawning, collected from the breeding tanks, and staged in hours post-fertilization, dpf or months post-fertilization (mpf), as previously described (Kimmel et al., 1995). The permits for all experiments were obtained from the Office of the Regional Government of Southern Finland, in agreement with the ethical guidelines of the European convention.

### Genotyping

At 3 dpf, larval genomic DNA was lysed after individual tail clippings were incubated in 50 μl lysis buffer (10 mmol/l Tris-HCl pH 8.3, 50 mmol/l KCl, 0.3% Tween 20 and 0.3% NP-40) at 98°C for 10 min, followed by incubation on ice for 2 min. Then, 1 μl Proteinase K (20 mg/ml) was added to remove protein, and the mixture was incubated at 55°C for at least 4 h. To inactivate Proteinase K, samples were incubated at 98°C for 10 min and quenched on ice.

To detect mutations, high-resolution melting curve acquisition and analysis was performed. Primers flanking the mutation site were designed using Primer-BLAST and were as follows: Forward, 5′-AATGACAGGAGCGCAAGTTT-3′ and Reverse, 5′-GTAAACCTCCTCATTCACCGTC-3′. High-resolution melt analysis (HRMA) was done on a LightCycler^®^ 480 instrument (Roche, Mannheim, Germany) using the following reaction mixtures: 1× LightCycler 480 HRMA master mix (Roche), 2 mmol/l MgCl_2_ and 0.15 μmol/l primer mixtures. The PCR cycling protocol was as follows: one cycle of 95°C for 10 min; 45 cycles of 95°C for 10 s, 60°C for 15 s, 72°C for 20 s and melting curve acquisition; one cycle of 95°C for 60 s and 40°C for 60 s. PCR products were denatured at 95°C for 60 s, renatured at 40°C for 60 s, and melted at 60-95°C with 25 signal acquisitions per degree. Melting curves were generated over a 65-95°C range. Curves were analyzed using the LightCycler^®^ 480 gene-scanning software (version 1.5) according to the manufacturer's instructions (Roche Diagnostics Ltd., Rotkreuz, Switzerland). To identify deviations of the curves indicative of sequence mutations, a three-step analysis was performed using the Gene Scanning program (Roche) as follows: (1) normalizing the raw melting-curve data by setting the initial fluorescence uniformly to a relative value of 100% and the final fluorescence to a relative value of 0%; (2) determining the temperature threshold at which the entire double-stranded DNA was completely denatured; (3) further analyzing the differences in melting-curve shapes (threshold setup 0) in order to cluster the melting curves with similar shapes into the same groups. Those with analogous melting curves were characterized as the same genotype. After genotyping, larval (6-10 dpf), juvenile (30-40 dpf) and adult (12 mpf) fish were raised and used in the experiments. All the comparisons were made between mutants and their WT siblings.

### Behavioral assays

The samples used in a particular behavioral test were not included in other tests at different developmental stages. All adult individuals in a behavioral evaluation were male siblings, and tests were repeated at least once with an independent biological replicate.

#### 24 h locomotion tracking

At 6 dpf, *mao* larvae of each genotype were tracked simultaneously for 24 h, with the light conditions following the regular light/dark cycle of the larvae (Puttonen et al., 2018). The trial was started at 12:00. The day and night activity was analyzed in 60 min bins by calculating the total distance moved. Larvae were individually tracked using the DanioVision (Noldus Information Technology, Wageningen, The Netherlands) system and EthoVision XT 13 software (Noldus Information Technology) in 48-well plates. The diameter of the wells was 12 mm.

#### Larval dark-flash response

The dark-flash response of larvae was evaluated at 6 dpf as described previously (Baronio et al., 2018). Briefly, after an initial 5 min of basic locomotor activity tracking, larvae were exposed to alternating 2 min periods of darkness and light, and with three periods of darkness in total. The locomotor activity was analyzed in 30 s and 1 s bins. Larvae were individually tracked in 48-well plates using the DanioVision system and EthoVision XT 13 software. This behavioral test was done between 12:00 and 16:00.

#### Larval acoustic/vibrational startle

This behavioral protocol has been described previously (Van Den Bos et al., 2017). Briefly, larvae (6 dpf) were transferred from Petri dishes to 48-well plates filled with 1 ml E3 medium. The protocol (lights on) consisted of 10 min acclimation, followed by ten acoustic/vibrational stimuli (DanioVision intensity setting 6) with a 20 s ISI. The variable of interest to show the startle response was maximum velocity (mm/s) with 1 s intervals, because the startle response is a short burst of activity best captured by this parameter. When subjects did not show a clear response to the first stimulus (values lower than 15 mm/s), they were discarded from analysis. Larvae were individually tracked using the DanioVision system and EthoVision XT 13 software. This behavioral test was done between 12:00 and 16:00.

#### Juvenile social interaction

At 30 dpf, an assessment of social contact behavior was carried out using a glass tank (9 cm length×5 cm height×7 cm width). Juvenile fish were separated into pairs of the same genotype and transferred to glass tanks with 150 ml water (water depth, 3 cm). Each member of the pairs was taken from different home tanks. We tracked the pairs' movements for 6 min, and results included the total duration in proximity (with proximity defined as when the distance between two larvae was ≤0.8 cm) Each group had 16 fishes for the data analysis using EthoVision XT 13 software.

#### Juvenile shoaling behavior

Zebrafish have the innate tendency to form tight groups of individuals (shoals), which is considered a form of social interaction that serves many purposes in nature, including foraging, avoiding predation and mating (Pitcher, 1986). The average nearest neighbor distance in a shoal significantly decreases with the age of zebrafish, and this change appears particularly robust between 30 dpf and 40 dpf (Buske and Gerlai, 2012). We evaluated this behavior, utilizing cohorts of four 40 dpf fish in a round white polyethylene plastic flat-bottomed container (12 cm height, 12 cm diameter) with 350 ml fish system water (5.0 cm depth). Before testing, fish were habituated for 5 min followed by video recording for 10 min with a camera at a fixed height (60 cm) from the top of the container. All videos were analyzed with EthoVision XT 13 software, using the default setting (the center-point detection of the unmarked animals). The mean of the interfish distance (defined as distance between the body center of every member of the shoal) was quantified from the average data from all trials (*n*=4 trials per genotype). The proximity duration (in s) was defined as the total duration of time a fish stayed close to the shoal fish (within 1 cm).

#### Adult social preference

This behavioral test has been described previously (Baronio et al., 2018). Briefly, the social preference of *mao*^+/+^ and *mao*^+/−^ adult animals was evaluated in an acrylic apparatus (29 cm length×19 cm height×29 cm width) divided by a transparent wall into two chambers, one of which was subdivided in two smaller compartments. A group of eight fish, serving as stimulus, was placed in one of the compartments, and the other compartment was filled with stones and plant imitations. The tested fish was placed in the other chamber, and the time spent in social, non-social and distal zones was measured with EthoVision XT 13 software.

#### Adult locomotor activity and thigmotaxis

Adult fish were individually tracked in separate cylindrical observation tanks (inner diameter 22 cm and water depth 8 cm), as described previously (Baronio et al., 2018). The fish had 10 min of habituation time in the tank before the tracking started. The swimming performance of the animals was automatically detected and tracked for 10 min by a digital video camera connected to a standard PC computer system running the EthoVision XT 13 software.

#### Novel tank diving test

Adult *mao*^+/+^ and *mao*^+/−^ zebrafish were transferred from their home tank into the novel tank (24 cm×14.5 cm×5 cm) with 11 of system water for behavioral observation and phenotyping (Chen et al., 2020). Novel tanks rested on a level, stable surface and were virtually divided into three equal portions (bottom, middle and top of the tank). Swimming behavior was recorded over a 6 min period, and the time spent in each part of the tank was measured using EthoVision XT 13 software. The degree of ‘bottom dwelling’ is generally interpreted as an index of anxiety-like behavior in zebrafish.

### RT-qPCR

Larval and adult zebrafish were killed in ice-cold water. Larval samples (ten pooled larvae per sample) and dissected adult brains were used for RNA extraction using an RNeasy mini Kit (Qiagen, Hilden, Germany). A total of 1.0 μg RNA was reverse transcribed using SuperScript™ III reverse transcriptase (Invitrogen, Carlsbad, CA, USA). RT-qPCR was done with a LightCycler 480 Real-Time PCR system and LightCycler 480 SYBR Green I Master kit (Roche Applied Science, Mannheim, Germany) as previously described (Chen et al., 2020). Primers for amplification were designed with Primer-BLAST (NCBI), and sequences are shown in Table S1. Results were evaluated with the LightCycler 480 Software version 1.5. Quantification was done by Ct value comparison, using the Ct value of ribosomal protein large subunit 13a (*rpl13a*) as the reference control.

### ISH

We used 4% paraformaldehyde (PFA)-fixed dissected larval brains and followed the protocol described by Thisse and Thisse (2008), with slight modifications, to perform ISH. A digoxigenin (DIG) RNA-labeling kit (Roche Diagnostics, Mannheim, Germany) was used to produce antisense DIG-labeled RNA probes. The specificity of the probes and clones have been described earlier (Chen et al., 2009, 2016; Kaslin et al., 2004; Peitsaro et al., 2007; Sundvik et al., 2011; Wang et al., 2006). The prehybridization and hybridization steps were performed at 60°C. Sheep anti-digoxigenin-AP Fab fragments (1:5000; Roche Diagnostics) were used to detect the ISH signals. Staining was performed with nitro blue tetrazolium and 5-bromo-4-chloro-3-indolyl-phosphate, and samples were incubated at room temperature in the dark. Stained samples were immersed in 80% glycerol, embedded between two cover glasses and analyzed under brightfield optics using a Leica DM IRB inverted microscope.

### Immunohistochemistry

Larvae were killed in ice-cold water and collected for overnight fixation in 4% 1-ethyl-3,3 (dimethyl-aminopropyl) carbodiimide (EDAC; Carbosynth, Berkshire, UK) or 2% PFA. The detailed protocol for immunohistochemistry and specificity of the antibodies have been described previously (Sallinen et al., 2009a). Primary antibodies were rabbit anti-histamine 19C (1:5000) (Panula et al., 1990), rabbit anti-5-HT antibody (1:1000; S5545, Sigma-Aldrich, St Louis, MO, USA) (Kaslin and Panula, 2001) and anti-Th1 monoclonal mouse antibody (1:1000; 22941, Immunostar, Hudson, WI, USA). For detection, the samples were incubated with Alexa Fluor-conjugated antibodies (Alexa Fluor anti-rabbit 488 and anti-mouse 568, Invitrogen) diluted 1:1000.

### Mao activity histochemistry

Mao enzyme activity histochemical assay was carried out as described (Sallinen et al., 2009b). Briefly, 4% EDAC-fixed 40 dpf brain samples were washed 2×10 min in PBS and then incubated in 0.05 M Tris-HCl buffer containing 0.08 g/l DAB, 1 g/l tyramine, 1 g/l peroxidase, 6 g/l NiSO_4_ for 2 h at room temperature.

### Microscopy and imaging

Brightfield images were taken with a Leica DM IRB inverted microscope with a DFC 480 charge-coupled device camera. *Z*-stacks were processed with Leica Application Suite software. Immunofluorescence samples were examined using a Leica TCS SP2 AOBS confocal microscope. The Alexa Fluor 488- and 568-labeled secondary antibodies were detected using a 488 nm argon laser and a 568 nm diode laser, respectively. Emission was detected at 500-550 nm and 560-620 nm, respectively. Stacks of images taken at 1.0 μm intervals were compiled, and the maximum intensity projection algorithm was used to produce final images with Leica Confocal software. Cell numbers were counted in each 1.0 μm optical slice using ImageJ 1.52b software (National Institutes of Health, Bethesda, MD, USA).

### Data and statistical analysis

Data shown are representative of a minimum of two independent biological replicates. Data were analyzed by unpaired, two-tailed Student's *t*-test, one-way analysis of variance (ANOVA) followed by Tukey's post hoc test or two-way multiple comparisons ANOVA followed by Tukey's post hoc test, and *P*<0.05 was considered statistically significant. Statistical analysis was performed by Prism version 7 (GraphPad Software, San Diego, CA, USA).

## Supplementary Material

Supplementary information
