# Supplementary material for: Abnormal brain development of monoamine oxidase mutant zebrafish and impaired social interaction of heterozygous fish
Source: Dis Model Mech. 2022 Feb 23;15(3):dmm049133. doi: 10.1242/dmm.049133 (PMC8891935; doi:10.1242/dmm.049133)
Supplement: Supplementary information [file dmm-15-049133-s1.pdf]

Table S1. qPCR primers

GENE	FORWARD PRIMER	REVERSE PRIMER
rpl13a	AGAGAAAGCGCATGGTTGTCC	GCCTGGTACTTCCAGCCAACTT
vmat2	TGCCTATTATCCCAAGTTACCTGT	TGAGGGCTCACAAAAGTAGGA
hdc	TTCATGCGTCCTCTCCTGC	CCCCAGGCATGATGATGTTC
th1	GACGGAAGATGATCGGAGACA	CCGCCATGTTCCGATTTCT
neurod1	ACACACCCTAGAGTTCCGAC	GTCCACGTCTCGTTCGTCTT
apoeb	AACGCCTGAACAAGGACACA	GTATGGCTGGAAACGGTCCT
mao	TGGTGGAGGTCAGGACGGTGA	GCTGGTTCCTCAGAGGCGGC
notch1a	AGAGCCGGATTCAGCGGTC	TTACAGGGACGTGGAGAACAAG
mecp2	ACGTCTACCTTATCAACCCAGA	CCTTCCACGTCCAGAGGG
shank3b	GGTTTTATTAGGGTTGTGAGGCCG	CCCAGGAGAGGTCCGAATACTGTC
gfap	GAAGCAGGAGGCCAATGACTATC	GGACTCATTAGACCCACGGAGAG
serta	ACAACCGATGGAACACTCCC	CAACACCTGCCGGACATAAA
hrh3	CGCCACCGTCCTTGGGAACG	GGGGATGCAAAACCCGCCGA

Disease Models & Mechanisms • Supplementary information
